# Early expansion of myeloid-derived suppressor cells inhibits SARS-CoV-2 specific T-cell response and may predict fatal COVID-19 outcome

**DOI:** 10.1038/s41419-020-03125-1

**Published:** 2020-10-27

**Authors:** Alessandra Sacchi, Germana Grassi, Veronica Bordoni, Patrizia Lorenzini, Eleonora Cimini, Rita Casetti, Eleonora Tartaglia, Luisa Marchioni, Nicola Petrosillo, Fabrizio Palmieri, Gianpiero D’Offizi, Stefania Notari, Massimo Tempestilli, Maria Rosaria Capobianchi, Emanuele Nicastri, Markus Maeurer, Alimuddin Zumla, Franco Locatelli, Andrea Antinori, Giuseppe Ippolito, Chiara Agrati

**Affiliations:** 1 National Institute for Infectious Diseases Lazzaro Spallanzani-IRCCS- Via Portuense, 292- 00149 Rome, Italy; 2 Immunotherapy Programme, Champalimaud Centre for the Unknown, Lisbon, Portugal; 3 I Med Clinic, University of Mainz, Mainz, Germany; 4 Division of Infection and Immunity, University College London, London, UK; 5 National Institute of Health Research Biomedical Research Centre, University College London Hospitals NHS Foundation Trust, London, UK; 6 Department of Pediatric Hematology and Oncology, Ospedale Pediatrico Bambino Gesù, Istituto di Ricovero e Cura a Carattere Scientifico, Sapienza, University of Rome, Rome, Italy

**Keywords:** Viral infection, Infection

## Abstract

The immunological mechanisms underlying the clinical presentation of SARS-CoV-2 infection and those influencing the disease outcome remain to be defined. Myeloid-derived suppressor cells (MDSC) have been described to be highly increased during COVID-19, however, their role remains elusive. We performed an in depth analysis of MDSC in 128 SARS-CoV-2 infected patients. Polymorphonuclear (PMN)-MDSC expanded during COVID-19, in particular in patients who required intensive care treatments, and correlated with IL-1β, IL-6, IL-8, and TNF-α plasma levels. PMN-MDSC inhibited T-cells IFN-γ production upon SARS-CoV-2 peptides stimulation, through TGF-β- and iNOS-mediated mechanisms, possibly contrasting virus elimination. Accordingly, a multivariate regression analysis found a strong association between PMN-MDSC percentage and fatal outcome of the disease. The PMN-MDSC frequency was higher in non-survivors than survivors at the admission time, followed by a decreasing trend. Interestingly, this trend was associated with IL-6 increase in non-survivors but not in survivors. In conclusion, this study indicates PMN-MDSC as a novel factor in the pathogenesis of SARS-CoV2 infection, and open up to new therapeutic options.

## Introduction

The ongoing COVID-19 pandemic due to the new coronavirus SARS-CoV-2 remains a global health emergency. As of 9 August 2020, there have been 19,462,112 COVID-19 cases with 722,285 deaths reported to the WHO (Ref: WHO Coronavirus Disease situation reports: https://www.who.int/emergencies/diseases/novel-coronavirus-2019/situation-reports/).

The clinical presentations of Covid-19 range from asymptomatic, mild, moderate to severe pneumonia and fulminant disease^1^.

The immunological mechanisms underlying the heterogeneous clinical expression of SARS-CoV-2 infection and those underlying factors influencing the clinical outcome remain to be defined. Tissue damage has been associated with excessive and uncontrolled immune activation and pro-inflammatory cytokines^2,3^. A massive infiltration of mononuclear cells has been detected in the lungs, with parallel low levels of hyperactive T cells in the peripheral blood^4^. Moreover, depletion of lymphocytes and increase of neutrophils in the peripheral blood are changes typically associated with an unfavorable disease course. In particular, it has been reported that the Lymphocyte/Neutrophil ratio is an independent risk factor of mortality for Covid-19 patients^5–7^. Notably, lymphocytes from patients with severe Covid-19 often present an exhausted phenotype^8,9^, and the macrophage activation syndrome has been described to occur in patients with respiratory failure^10^. Together, these data indicate that SARS-CoV-2 infection associated with excessive and dysregulated immune activation, and massive migration of cells to the infected tissue, to control viral replication, could contribute to tissue damage.

During evolution, immune system has developed regulatory mechanisms able to control excessive inflammation/activation, such as the induction of inhibitory receptor expression, production of specialized anti-inflammatory cytokines, and expansion of regulatory cells. However, their role during SARS-CoV-2 infection is poorly understood. We recently reported the expansion of myeloid-derived suppressor cells (MDSC) during SARS-CoV-2 infection, correlated with inflammatory milieu^11,12^. Whether MDSCs play a beneficial anti-inflammatory and/or a detrimental immune suppressive role during COVID-19 has not yet been elucidated.

MDSC, defined in humans as CD11b +CD14−CD33+CD15+ and HLADR-low (polymorphonuclear MDSCs), or CD11b+CD14+CD33+ and HLA-DR-low (monocytic MDSCs), are known to have the remarkable ability to reduce overt inflammation and suppress T-cell responses through several mechanisms, including inducible nitric oxide synthase (iNOS), arginase-1(Arg-1), nicotinamide adenine dinucleotide phosphate oxidase (NOX2), and transforming growth factor beta (TGF-β)^13–16^.

In the present work, we provide evidence that early PMN-MDSC expansion inhibits SARS-CoV-2 specific T-cell responses, and might predict fatal outcome.

## Materials and methods

### Sample size calculation

Sample size was calculated by using a significance level (alpha) of 0.050, and performing a one-sided two-sample equal-variance *t*-test. Group sample sizes of 96 (no ICU 75%) and 32 (ICU 25%) achieve 95% power to reject the null hypothesis of equal means of MDSC in the two groups, when the population mean difference is more than 15.0 (standard deviation = 22 for both groups). For the follow up analysis, group sample sizes of 59 (survivors 75%) and 19 (non survivors 25%), for a total of 78 patients achieve 90% power to reject the null hypothesis when the population mean difference is more than 17.0.

### Study population

Patients with confirmed SARS-CoV-2 infection (*n* = 128) were treated at the National Institute for Infectious Diseases (INMI) “LazzaroSpallanzani” (Rome, Italy). All patients were symptomatic, ranging from mild to severe (requiring intensive care unit admission, *n* = 32). Median age was 63 years (IQR 53–75), 88 (68.8%) were males. Healthy individuals (HD, *n* = 30) were included as controls. Patients were recruited in the study at the time of hospital admission (within 2 weeks from symptoms onset). A group of patients (*n* = 78) were followed weekly, until 5 weeks after admission. At the time of manuscript preparation, 59 patients recovered and were discharged (survivors), and 19 died (non-survivors).

The study was approved by the Institutional Review Board of the INMI “LazzaroSpallanzani” (approval number: 9/2020) and signed written informed consent was obtained from all patients.

### Plasma cytokine levels

Plasma samples were obtained from peripheral blood, after speed centrifugation for 10 min at 2000 rpm and immediately stored at −80 °C. IL1-β, IL-6, IL-8, TNF-α, and TGF-β were measured in plasma samples by using customized automated ELISA assays (ELLA microfluidic analyzer, Protein Simple, Bio-Techne, USA).

### Peripheral blood mononuclear cells and PMN-MDSC isolation

PBMCs were isolated from heparin treated whole blood by density gradient centrifugation (Lympholyte-H, cat. CL5020; Cederlane, USA.) PBMCs were suspended in RPMI 1640 (cat. 15040- CV, Corning Incorporated, USA) supplemented with 10% heat-inactivated fetal bovine serum (FBS) (cat. EU S028877, EuroClone, Italy), penicillin/streptomycin solutions,100X (cat. 30-002-CL, Corning Incorporated, USA), 2 mmol/L L-glutamine (cat. M11-004, Corning Incorporated, USA), and 10 mmol/L HEPES buffer (N-2- hydroxyethylpiperazine-N-2-ethane sulfonic acid, cat. 15630106, Gibco, USA), hereafter termed R10.

Magnetic cell isolation technology was used to isolate the PMN-MDSC cells from PBMCs. Positive selections were performed by CD15 direct magnetic labeling (cat. 130-046-601, MiltenyiBiotec, Germany), according to the manufacturer’s procedure. Purity and recovery was >90% and were verified by flow-cytometry (data not shown).

### Flow-cytometry

MDSC frequency was evaluated by staining PBMCs with customized Duraclon tubes, (CD11b FITC, HLA-DR ECD, CD14 PC5.5, CD33 PC7, CD45KrO, CD80 APC, DRAQ7, CD56 APC-alexa750, CD19 APC-alexa750, CD3 APC-alexa750, CD15 Pacific Blue, cat. B74697, Beckman Coulter), following manufacturer’s procedures. Data were acquired by CytoFlex flow-cytometer (Beckman Coulter, USA). Data were analyzed by CytExpert or Kaluza software (Beckman Coulter, USA).

### Proliferation assay

PBMC were isolated from SARS-CoV-2+ patients. PBMCs and PMN-MDSC-depleted PBMCs (Depleted) were stained with CFDA-SE (Vibrant CFDA SE cell tracer kit, cat. V1288, Invitrogen, USA) following manufacturer’s procedures. PBMCs, PMN-MDSC depleted PBMCs, and PMN-MDSC depleted PBMC plus PMN-MDSC (1/4 ratio) were cultured in 48-well flat plates (500,000 cells/well in R10), stimulated with Staphylococcus enterotoxin B (SEB, 800 ng/ml, cat. S4881, Sigma Aldrich, USA) and maintained at 37 °C in humidified air with 5% CO^2^. After 4 days, cells were stained with anti-CD3 APC (cat. 555335, BD Biosciences, USA), and acquired by CytoFlex flow cytometer (Beckman Coulter, USA). Data were analyzed by CytExpert software (Beckman Coulter, USA).

### SARS-CoV-2 specific T-cell response

SARS-CoV-2 specific T-cell response was performed by stimulating PBMC, PMN-MDSC depleted PBMCs, and Depleted PBMC plus PMN-MDSC (1/4 ratio) with a pool of Spike and Nucleocapside derived peptides (1 μg/ml, Peptide&Elephants GmbH, Germany) for 4 days. Phytohaemagglutinin (PHA, cat. 11082132001, Sigma Aldrich, USA) was used as control for T cell activity. The IFN-γ release was evaluated by using a direct, sandwich chemiluminescence immunoassay (CLIA), and analyzed by LIAISON® XL analyzer, (Diasorin). Results were reported in International Units per mL (IU/mL).

Where indicated, PBMCs, PMN-MDSC depleted PBMCs, and Depleted PBMCs plus MDSC (ratio 1:4), were treated with N(ω)-hydroxy-nor-L-arginine (0.5 mM, cat. 399275, Calbiochem, Germany), neutralizing anti-human TGFβ antibody (10 μg/ml, cat. AB 101 NA, R&D Systems, USA), or L-N^G^-nitro arginine methyl ester (1 mM, cat.483125, Calbiochem, Germany). The production of IFN-γ was evaluated by ELISA (Quantikine ELISA, cat. PDIF50C, R&D Systems, USA).

### Quantitative PCR

Total RNA was extracted from PMN-MDSC cells using Direct-zol™ RNA MiniPrep KIT (cat. R1055, Zymo Research Corp., USA) according to the manufacturer’s instructions. For RT-PCR analyses, single-stranded cDNA was obtained by reverse transcription of 1 μg of total RNA using AMV-reverse transcriptase (cat. M5108, Promega, MI, Italia). ArgI, i-NOS and TGF-β mRNA were analyzed by quantitative PCR by using the following primers: Arginase1 F: 5′-CGCCAAGTCCAGAACCATAG-3′ R: 5′-TCCCCATAATCCTTCACATCAC-3′; iNos F: 5′-AGATAAGTGACATAAGTGACCTG-3′ R: 5′-CATTCTGCTGCTTGCTGAG-3′; TGF-β F: 5′-GACATCAACGGGTTCACTAC-3′ prime R: 5′-GTGGAGCTGAAGCAATAGTT-3′. qPCR was performed FastStart Essential DNA Green Master (cat. 06402712001, Roche Life Science, USA) according to the manufacturer’s instructions. The expression levels were normalized to the β2-microlobulin level (β2-microlobulin F: GAGTATGCCTGCCGTGTG, R: AATCCAAATGCGGCATCT) using the equation 2−ΔCt. All qPCR reactions were performed in a Corbett 212 Rotor-gene6000 Real-Time PCR System.

### Statistical analysis

GraphPad Prism version 4.00 for Windows (GraphPad Software) and STATA 15.1 were used to perform statistical analyses. The non-parametric Kruskal-Wallis with Dunn’s post test, or the Mann–Whitney test were used to compare continuous variables. Correlations were evaluated with the non-parametric Spearman test. To evaluate the performance of PMN-MDSC frequency as candidate biomarker in discriminating between survivors and non-survivors, a receiver operating characteristic (ROC) curve analysis was performed. Cox regression analysis was used to estimate hazard ratio (HR) and 95%CI of death adjusted for gender and age at admission. A *p* value < 0.05 was considered statistically significant.

## Results

### Inflammation promotes PMN-MDSC expansion in COVID-19 patients

**Fig. 1:**
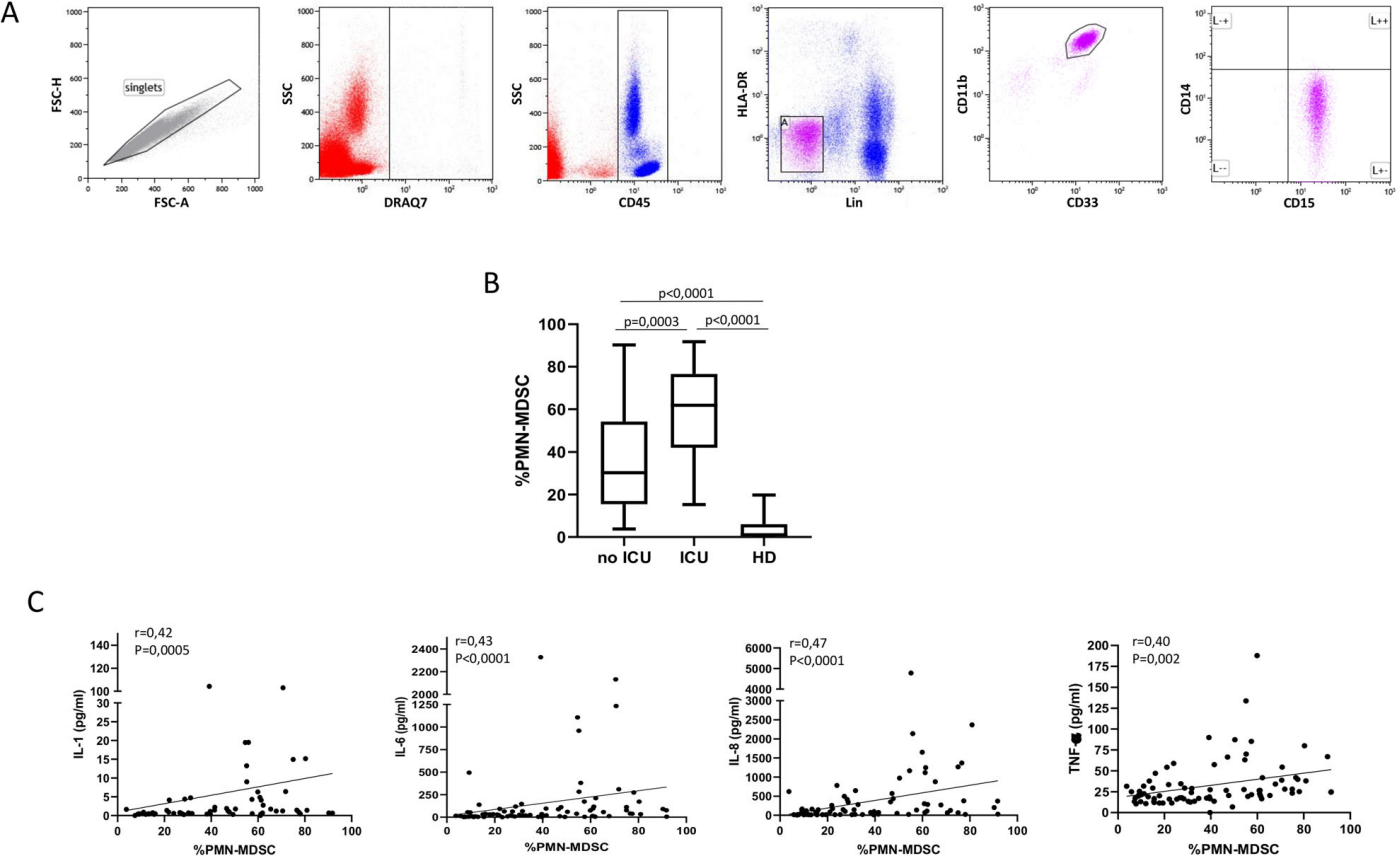
PMN-MDSC are increased in SARS-CoV-2 infected patients requiring ICU admission. **A** Representative plots of the adopted gating strategy to identify MDSC. Doublets were excluded in the FSC-H/FSC-A dot plot. Live cells were selected as DRAQ7−. In the immunological plot (SideScatter (SSC)/CD45), the CD45+ cells were gated, followed by gating on Lin−(CD3−CD19−CD56−)/HLA-DRlow/− cell. Cells were then selected as CD33+CD11b+, and the expression of CD15+ and CD14− was evaluated. PMN-MDSC frequency was calculated in the immunological gate (CD45+ cells). **B** Frequency of PMN-MDSC at the admission time in 96 patients who did not require ICU admission, in 32 who did require ICU admissions, and in 30 healthy donors (HD). Results are shown as Box and Whiskers. Kruskal–Walliswith Dunn’s post test was applied. **C** Correlations between PMN-MDSC frequency and inflammatory cytokines in 78 COVID-19 patients. Non-parametric Spearman correlation was applied, and *p* < 0.05 was considered significant.

At the admission time, we found a higher frequency of the PMN-MDSC cell population (identified as Lin−, HLA-DR−, CD11b+, CD33+, CD15+, CD14−; Fig. 1A) in 128 hospitalized COVID-19 patients requiring or not requiring intensive care unit (ICU) compared to HD (Fig. 1B). A higher percentage of PMN-MDSC was found in patients requiring ICU compared to patients who did not required ICU care (Fig. 1B). We did not detect monocytic-MDSC in COVID-19 patients or in HD (data not shown). Group sample sizes of 96 (no ICU 75%) and 32 (ICU 25%) achieve 95% power to reject the null hypothesis of equal means of MDSC in the two groups, when the population mean difference is more than 15.0 (standard deviation = 22 for both groups).

It is well know that the inflammatory cytokines play a central role in inducing the expansion of MDSC^17^. We quantified, therefore, the IL-1β, IL-6, IL-8, and TNF-α plasma level, and found a significant direct correlation between PMN-MDSC frequency and such inflammatory cytokines (Fig. 1C).

### PMN-MDSC inhibit SARS-CoV-2 specific T-cell response

**Fig. 2:**
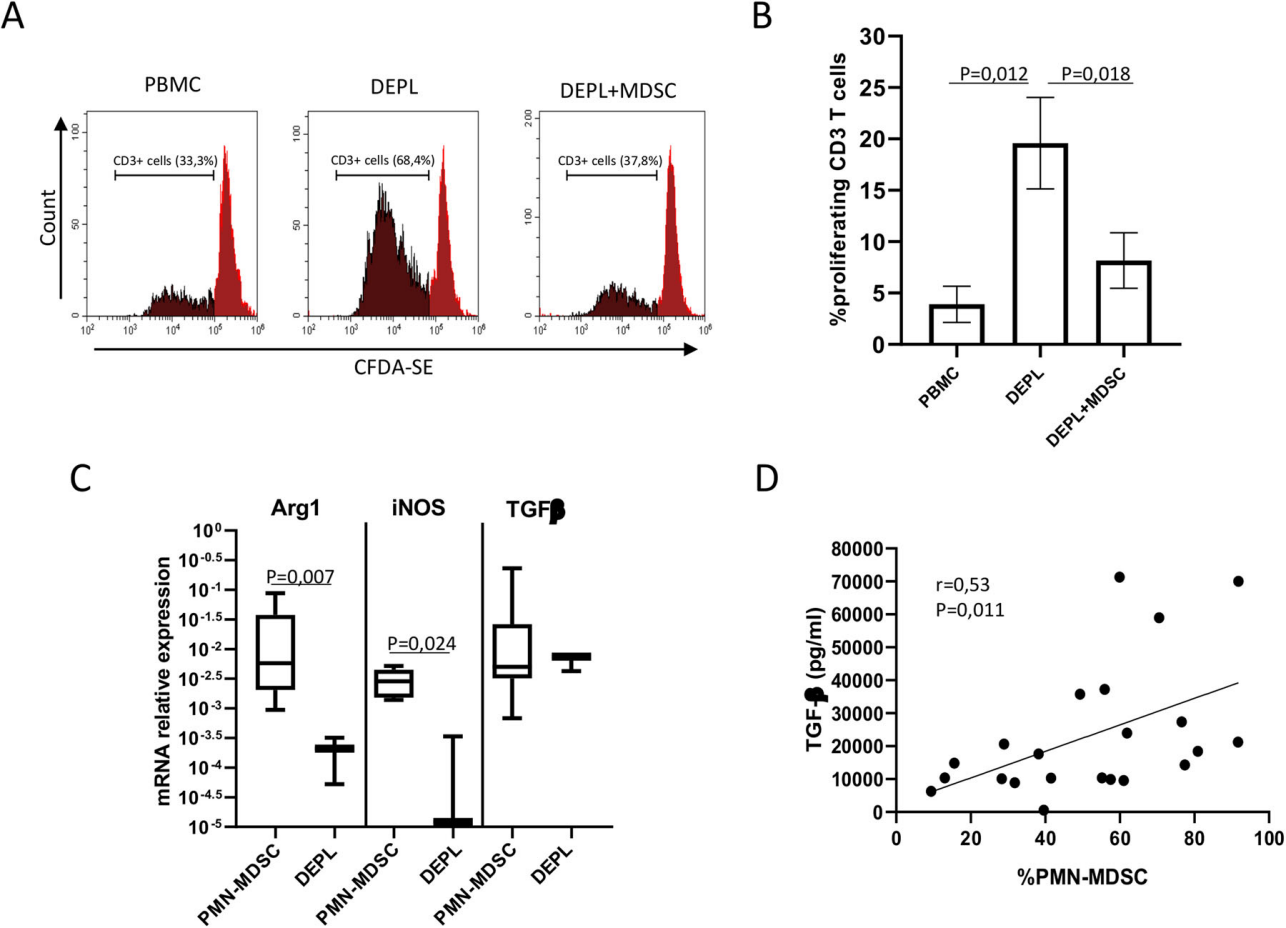
PMN-MDSC inhibit T cell proliferation. **A** Representative flow-cytometry histogram plots of the proliferation rate of CD3+ cells upon stimulation with SEB. **B** Percentage of CD3+ proliferating cells in PBMC, PMN-MDSC depleted PBMC (DEPL), and DEPL + PMN-MDSC (1/4 ratio) from 5 patients. Results are shown as mean ± SEM. Paired *T* test was performed. *p* < 0.05 was considered significant. **C** mRNA of TGF-β, ArgI, and iNOS in purified PMN-MDSC from 10 COVID-19 patients. Results are shown as Box and Whiskers. **D** Correlation between PMN-MDSC and plasmatic TGF-β in 22 COVID-19 patients. Non-parametric Spearman correlation was applied, and *p* < 0.05 was considered significant.

In order to evaluate the suppressive function of PMN-MDSC, we first analyzed the ability of these cells to inhibit the proliferation of CD3+ T cells. We performed PMN-MDSC isolation from PBMC by using CD15 coated microbeads, indeed the CD15+ cells were HLA-DR-, CD11b+`, CD33+ (Supplementary Fig. 1), thus matching the PMN-MDSC phenotype definition. We stimulated either (i) whole PBMC, (ii) PMN-MDSC-depleted (CD15−) PBMC (DEPL), and (iii) PMN-MDSC-depleted PBMCs plus PMN-MDSC (1/4 ratio) (DEPL + MDSC) with SEB, and the proliferation of T cells was tested after four days of culture. We found that PMN-MDSC depletion significantly increase the proliferation rate of CD3+ cells; when PMN-MDSC were added again at 1–4 ratio, the proliferation returned to a similar level as whole PBMC (Fig. 2A, B). To characterize the suppressive ability of PMN-MDSC, the expression of ArgI, TGF-β, and iNOS mRNAs were evaluated in purified PMN-MDSC and DEPL from ten COVID-19 patients. Results showed that PMN-MDSC expressed higher level of Arg1 and iNOS mRNA compared to DEPL (Fig. 2C). Differently, TGF- β was similarly expressed in PMN-MDSC and DEPL. We evaluated the plasmatic level of TGF-β, and found a direct correlation between PMN-MDSC frequency and TGF-β protein in plasma (Fig. 2D), indicating that PMN-MDSC significantly contribute to the production and release of TGF-β.

**Fig. 3:**
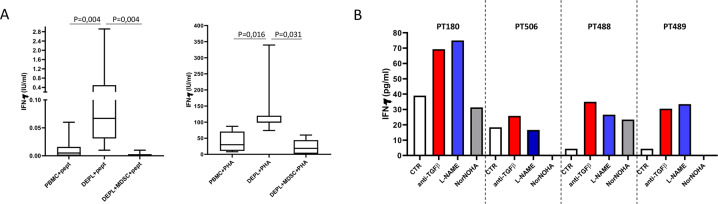
PMN-MDSC inhibit SARS-CoV-2 specific T cell response through TGF-β and NO release. **A** IFN-γ production from PBMC, PMN-MDSC depleted PBMC (DEPL), and DEPL + PMN-MDSC (1/4 ratio) from 11 patients upon stimulation with SARS-CoV-2 peptides or with PHA. Results are shown as Box and Whiskers. Kruskal–Walliswith Dunn’s post test was applied. **B** IFN-γ production from PBMC from 4 patients upon stimulation with SARS-CoV-2 peptides (CTR) and neutralizing anti-human TGF-β, Nor-NOHA, or L-NAME.

We then evaluated the impact of PMN-MDSC on SARS-CoV-2 specific T cell response. The stimulation of PBMC with SARS-CoV-2 peptides induced only a very low IFN-γ release from T cells. In contrast, PMN-MDSC depletion was able to significantly improve the antigen-specific T-cell response, defined by the increase of IFN-γ production upon SARS-CoV-2 peptide stimulation. This specific T-cell response was switched off when PMN-MDSC were added again (1:4 ratio) as shown by the complete inhibition of the IFN-γ release (Fig. 3A). Similar results were obtained by using PHA that trigger the CD3 complex (Fig. 3A). To define the possible mechanisms used by PMN-MDSC to inhibit SARS-CoV-2 specific T-cell response, we stimulated PBMC with SARS-CoV-2 peptides in the presence of neutralizing anti-TGF-β antibody, an inhibitor of ArgI (N(ω)-hydroxy-nor-L-arginine, Nor-NOHA), and an inhibitor of iNOS (L-N^G^-nitro arginine methyl ester, L-NAME). Figure 3 panel B shows that anti-TGF-β and L-NAME treatments were able to increase the capacity of T cells to produce IFN-γ in 100% (four out of four), and 75% (three out of four) patients, respectively, indicating that TGF-β and iNOS were involved in the PMN-MDSC mediated suppressive activities.

### High PMN-MDSC frequency is associated with SARS-CoV-2 fatal infection

**Fig. 4:**
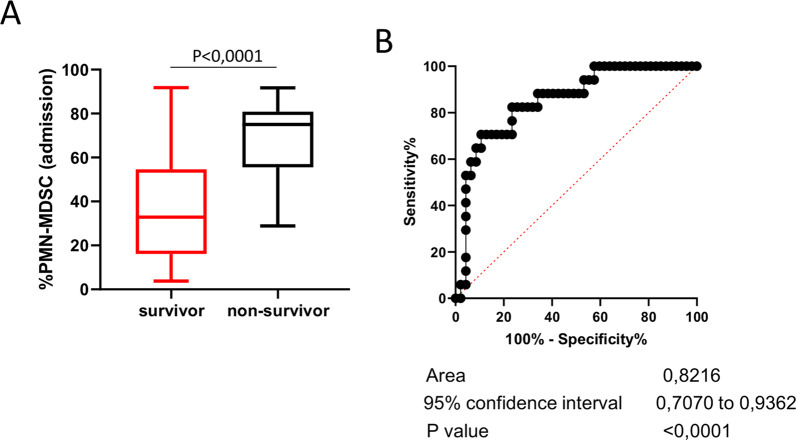
High PMN-MDSC frequency associate with COVID-19 fatal outcome. **A** PMN-MDSC frequency at the time of admission grouped in survivors (*n* = 59) and non-survivors (*n* = 19). Results are shown as Box and Whiskers. Mann–Whitney test was applied, and a *p* value < 0.05 was considered significant. **B** Receiver operating characteristic (ROC) curve for the PMN-MDSC frequency as marker of COVID-19 fatal outcome.

T-cell response is central in the adaptive immune-mediated elimination of pathogens. Since we showed the detrimental impact of PMN-MDSC on T-cell response to SARS-CoV-2, we wondered whether PMN-MDSC frequency could be predictive of disease outcome. We retrospectively grouped patients into different groups, namely, those who recovered (survivors, *n* = 59) and those who succumbed to the disease (non-survivors, *n* = 19), and analyzed the relative PMN-MDSC frequency at the time of admission. We found a significantly higher frequency of PMN-MDSC in the non-survivor compared with the survivor group (Fig. 4A), suggesting that PMN-MDSC percentage could be predictive of disease outcome. To validate this hypothesis, we performed a ROC analysis and found that PMN-MDSC frequency at the admission could distinguish between survivors and non-survivors (Fig. 4B; area under the curve 0.82, *p* < 0.0001), identifying a cut off value of 54.91% (77.8% and 78% of sensibility and specificity, respectively). This data were further confirmed by estimating the HR of death for MDSC in a Cox regression analysis adjusting for age and gender: the increase of 1% in PMN-MDSC frequency was independently associated with an augmentation of 3% of risk of fatal outcome (Table 1).

**Table 1:** Cox regression analysis.

	HR	95%CI		*p*
MDSC, for each additional unit	1.03	1.01	1.05	0.005
Age, for each additional year	1.02	0.99	1.06	0.179
Gender, Female vs Male	0.78	0.27	2.24	0.644

### PMN-MDSC frequency kinetic

**Fig. 5:**
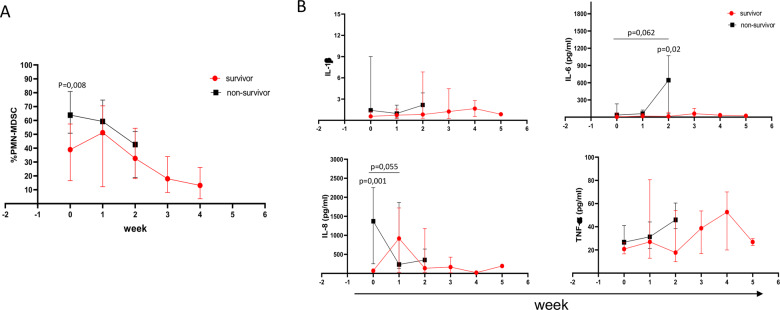
PMN-MDSC and inflammatory cytokines kinetic. **A** PMN-MDSC frequency in survivors (red line) and non-survivor (black line) patients was monitored weekly. Results are expressed as median and IQR. Mann–Whitney or Friedman test were applied to compare the PMN-MDSC frequency between the two groups at different time points or to compare different time points, respectively. A *p* value < 0.05 was considered significant. **B** Kinetic of the indicated cytokines in survivors (red line) and non-survivor (black line) patients. Results are expressed as median and IQR. Mann–Whitney or Friedman test were applied to compare the cytokine levels between the two groups at different time points or to compare different time points, respectively. A *p* value < 0.05 was considered significant.

To evaluate whether PMN-MDSC frequency persisted high during the time of infection, a subgroup of patients was monitored weekly until discharged/death. While at the admission survivors and non-survivor did differ in PMN-MDSC frequency, this was not observed during the longitudinal follow-up: both groups, survivors and non-survivors, showed a decreasing trend of PMN-MDSC percentage after admission (Fig. 5A).

Since a correlation between PMN-MDSC and inflammatory cytokines was observed, we analyzed IL-1b, IL-6, IL-8, and TNF-a at the same time points, and found that, accordingly with PMN-MDSC frequency, the plasma IL-8 level was higher in non-survivors than in survivors at the admission time, and decreased to level comparable to survivors at later time points (Fig. 5B). Interestingly, plasma IL-6 levels in the group of non-survivors increased after two weeks from admission, resulting significantly higher than in survivors (Fig. 5B). We did not observe any significant difference for TNF-α and IL-1β.

## Discussion

We identified the PMN-MDSC frequency as an early marker of COVID-19 fatal outcome involved in the inhibition of SARS-CoV-2 specific T-cell response. The increased proportion of circulating MDSCs, mainly described in cancer, is attracting great interest in infectious diseases^18–22^ Although MDSC typically arise in in peripheral tissues to dampen inflammation and inflammation-induced tissue damage, a detrimental role of this cell population in cancer and infectious diseases has been widely reported^21,23,24^. We recently found an expansion of PMN-MDSC in the peripheral blood of COVID-19 patients compared to HD, in particular in patients experiencing severe disease^11,12^. We observed an expansion of PMN-MDSC in non-ICU and ICU requiring patients, with the latter group presenting with a higher PMN-MDSC frequency. Following the recommendation for MDSC nomenclature and characterization^25^, the cells phenotypically here identified as PMN-MDSC were confirmed to act as suppressive cells, since they were able to effectively inhibit T-cell proliferation.

It has been proposed that inflammation promotes MDSC expansion and up-regulation of their suppressive capability^17^. In COVID-19 patients, a hyper-inflammation has been described^2,26^ that possibly induced PMN-MDSC differentiation and expansion. Indeed, we observed a correlation between PMN-MDSC frequency and the plasma level of IL-1β, IL-6, IL-8, and TNF-α. Interestingly, patients who will ultimately succumbed to SARS-CoV-2 infection showed a higher frequency of PMN-MDSC at the time of admission as compared to patients who ultimately survived, suggesting that PMN-MDSC frequency can be used as a prognostic marker of disease outcome. The non-survivors PMN-MDSC frequency matched with a high IL-8 level at the admission time, which decreased at later time points, in parallel to the reducing trend of PMN-MDSC. Among the pro-inflammatory mediators, IL-8 has been linked to the recruitment of MDSCs^27,28^, suggesting that IL-8 could have a major role in PMN-MDSC maintenance. Of note, in non-survivors a significant increase of IL-6 was found after 2 weeks from admission, suggesting that, the decrease of PMN-MDSC frequency could account for IL-6 rise. High IL-6 levels has been recently associated with fatal COVID-19 infection^29^, suggesting that immune suppression, possibly mediated by expanded MDSC, could be highly beneficial in reducing inflammation.

PMN-MDSC from COVID-19 patients expressed typical mRNA associated with MDSC suppressive functions such as ArgI, TGF-β, and iNOS, providing evidence that these cells exhibited a highly suppressive potential. Moreover, PMN-MDSC frequency correlated with the plasma level of TGF-β, indicating that PMN-MDSC contribute to TGF-β release, which, in turn, may act as a potent enhancer of the MDSC inhibition function^30^. L-Arginine, nitric oxide, and TGFβ pathways have been reported to play a major role in the regulation of innate and adaptive immune response^31–33^. In particular, these molecules influence human T-cell proliferation, differentiation, and survival^34^. The importance of the specific T cells has been demonstrated for virus clearance, for limiting tissue damage, and dampening overactive innate immune responses^35–37^. Two recently published studies demonstrated a SARS-CoV-2 specific T-cell response in convalescent/recovered patients^38,39^. However, timing, composition, magnitude, persistence, and protective role of T-cell response during COVID-19 are still to be fully elucidated. Our data showed a low SARS-CoV-2 specific T-cell response during COVID-19, defined by IFN-γ production, which was increased by depleting MDSC, suggesting that the low T cell responses are due to in vivo suppressive activity, not associated with the absence of antigen-specific T cells. We also found that TGF-β and iNOS produced by PMN-MDSC mediated their suppressive activity. PMN-MDSC from COVID-19 patients also inhibit PHA-induced IFN-γ production, indicating a powerful suppressive activity on T cells.

The suppressive potential of expanded MDSC can inhibit also NK-cell function^16^. The observation of reduction in the granzime A content of NK cells in patients with COVID-19^40^ could therefore also be associated with the MDSC-induced suppression of NK activity. We have also reported a negative correlation between PMN-MDSC frequency and perforin expressing NK cells^11^, consolidating that PMN-MDSC are indeed involved in inhibiting the cytotoxic potential of NK cells.

The ROC analysis confirmed the detrimental role of PMN-MDSC during COVID-19, as their high frequency at early stage of the disease correlated with fatal disease outcome. A logistic regression analysis showed that patients with an early increased PMN-MDSC frequency had a high risk of mortality after adjustment for other cofounders. Altogether, our data indicate that a high PMN-MDSC frequency, even if reduces inflammation, can inhibit both adaptive and innate anti-viral immune response, thus preventing virus elimination and ultimately patients’ recovery.

In conclusion, despite the low number of patients analyzed in this report, this explorative study indicates new biologically and clinically relevant factors in the pathogenesis of SARS-CoV2 infection, namely the MDSC and TGF-β-mediated suppression of virus specific T cell function. Our data also highlights the rational for a possible use of therapeutic approaches focused on reducing MDSC number/function, thereby increasing anti-virus directed T-cell responses as a viable therapeutic approach for patients with cancer^41,42^.

## Supplementary information

supplentary figure
