# Supplementary figures and images for: Early expansion of myeloid-derived suppressor cells inhibits SARS-CoV-2 specific T-cell response and may predict fatal COVID-19 outcome
Source: Cell Death Dis. 2020 Oct 27;11(10):921. doi: 10.1038/s41419-020-03125-1 (PMC7590570; doi:10.1038/s41419-020-03125-1)

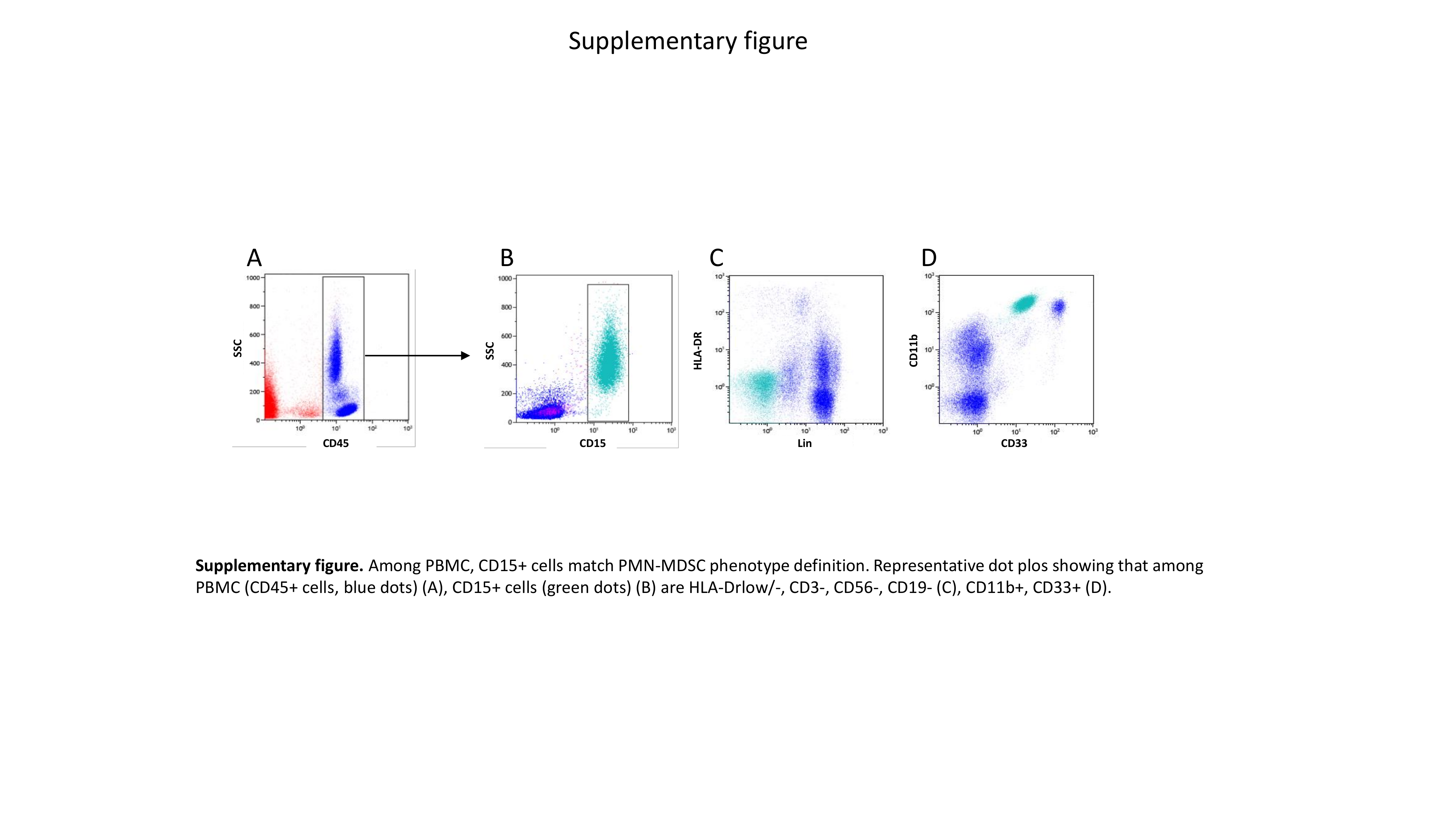

Supplement: Supplementary file 1 — supplentary figure [file 41419_2020_3125_MOESM1_ESM.tif]
